# Chiari III malformation with ruptured encephalocele: a case report and review of literature

**DOI:** 10.1097/MS9.0000000000003852

**Published:** 2025-09-16

**Authors:** Nischal Soti, Amit Bahadur Pradhanang, Prabhat Jha, Bipesh Bolakhe, Chahana Thapa, Bidhan Nepal

**Affiliations:** a Department of Neurosurgery, Tribhuvan University Teaching Hospital, Kathmandu, Nepal; b Maharajgunj Medical Campus, Institute of Medicine, Tribhuvan University, Kathmandu, Nepal

**Keywords:** case report, Chiari malformation type III, occipital encephalocele, syringomyelia

## Abstract

**Introduction and Importance:**

Chiari malformation type III (CM III) is the rarest and most severe form of Chiari malformations, involving herniation of the cerebellum and/or brainstem through an occipital or high cervical encephalocele. CM III is associated with severe neurological impairment and a poor prognosis due to extensive brain malformations. Surgical repair is the primary treatment to prevent complications like meningitis or encephalocele rupture.

**Case Presentation:**

A 2-month-old female baby presented with a ruptured occipital encephalocele and cerebrospinal fluid leakage. The swelling had been present since birth. She was born at term via emergency cesarean section to a primigravida mother who did not take antenatal folic acid. MRI revealed CM III. Surgery was initially delayed due to baby’s low birth weight. After rupture, emergency excision and repair were performed successfully.

**Case Discussion:**

CM III was characterized by herniation of brain structures through a high cervical or low occipital encephalocele. It accounts for less than 1% of cases and often presents at birth with neurological impairments and associated anomalies such as hydrocephalus and syringomyelia. Postnatal MRI shows herniated brain tissue and related abnormalities. Surgical repair is the main treatment, especially urgent if the encephalocele ruptures, to prevent infection and further complications

**Conclusion:**

CM III is a rare and severe birth defect where brain tissue protrudes through an opening in the skull, often causing serious neurological problems. Early diagnosis with imaging and prompt surgery, especially if the protrusion ruptures, are crucial to prevent complications.

## Introduction

Chiari malformation represents a spectrum of congenital abnormalities affecting the hindbrain, involving structural disruptions in the cerebellum, brainstem, and skull base. Type III is the least common and most severe variant, comprising less than 1% of all cases^[1]^. It is characterized by the protrusion of the cerebellum and/or brainstem through an encephalocele located in the occipital or high cervical region and is frequently associated with additional anomalies such as hydrocephalus, syringomyelia, and abnormalities of the corpus callosum. Additional variations may include scalloping of the petrous portion of the temporal bone and the clivus, absence or excessive growth of the cerebellum, displacement of the cerebellum around the brainstem, absence of the corpus callosum, defects in the posterior arch of the atlas, and absence of the falx cerebri^[2]^.

**HIGHLIGHTS**

- CM III is the rarest and most severe Chiari malformation.
- This case involves a ruptured encephalocele with CM III, a very rare presentation.
- Imaging showed cerebellar and brainstem herniation with hydrocephalus.
- Early surgery and team approach were key to stabilizing the patient.
- This report adds to global data on ruptured CM III encephaloceles.

Unlike the more common Chiari malformation types I and II, Chiari malformation type III (CM III) is frequently associated with significant neurological impairment and poor prognosis due to the degree of neural tissue displacement and associated brain malformation^[3]^. Newborns diagnosed with CM III frequently experience respiratory failure, difficulties with swallowing, increased muscle tone, or muscle weakness. Because of the severe respiratory problems linked to this condition, the outlook is generally considered very poor^[4]^. The primary treatment for encephalocele in patients with CM III is surgery, aimed at preventing meningitis or encephalocele rupture^[5]^.

We present a case of a ruptured encephalocele – a neurosurgical emergency due to the increased risk of cerebrospinal fluid (CSF) leakage, infection, and potential neurological deterioration – and its management at our hospital through excision and repair of the encephalocele.

This case report is written in accordance with SCARE guideline^[6]^.

## Case presentation

**Figure 1.**
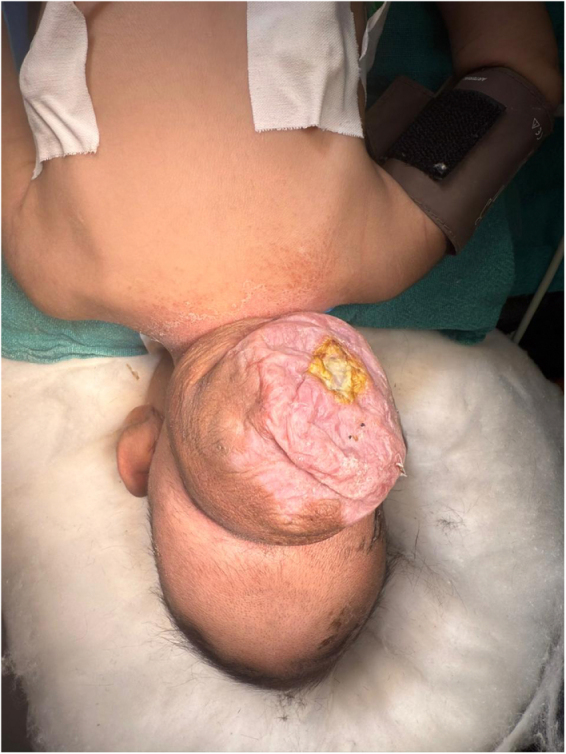
Image showing an irregularly shaped, smooth-surfaced swelling measuring approximately 10 × 9 cm, with overlying skin appearing thinned.

A 2-month-old female baby was admitted to our center with the chief complaint of rupture of an occipital encephalocele accompanied by clear discharge. Swelling had been present over occipital region since birth. The swelling was irregular in shape, had a smooth surface, and the overlying skin appeared thin (Fig. 1). On examination, the swelling measured 10 × 9 cm. There was transillumination, impulse on crying, and no bulging of the anterior fontanelle.

The baby was born to a primigravida mother at 38 weeks and 6 days of gestation through emergency cesarean section with no history of consanguinity. The mother underwent regular antenatal checkups but did not take antenatal folic acid. There was no history of use of teratogenic drugs, radiation exposure, or evidence of maternal infection during pregnancy. No abnormality was detected on antenatal scans. However, a mass protruding from the occipital region of the head was noted at birth.

The baby cried immediately after birth with an appearance, pulse, grimace, activity, respiration (APGAR) score of 6/10 and 7/10 at 1 and 5 minutes of life, respectively. At birth, she had notable features such as microcephaly, low-set ears, a depressed nasal bridge, and bluish discoloration on the dorsum of the right foot. A neurological consultation was obtained, and MRI was performed. The findings revealed inferior descent of the cerebellar tonsils and obex with occipital encephalocele and a syrinx in the spinal cord from C5 to L2 vertebral level – features suggestive of CM III. The echocardiogram revealed a patent foramen ovale with a left to right shunt. At birth, due to the baby’s low birth weight, no immediate neurosurgical intervention was performed, and the surgery was deferred until she reached 5 kg and was discharged with a follow-up plan for surgical management.

**Figure 2.**
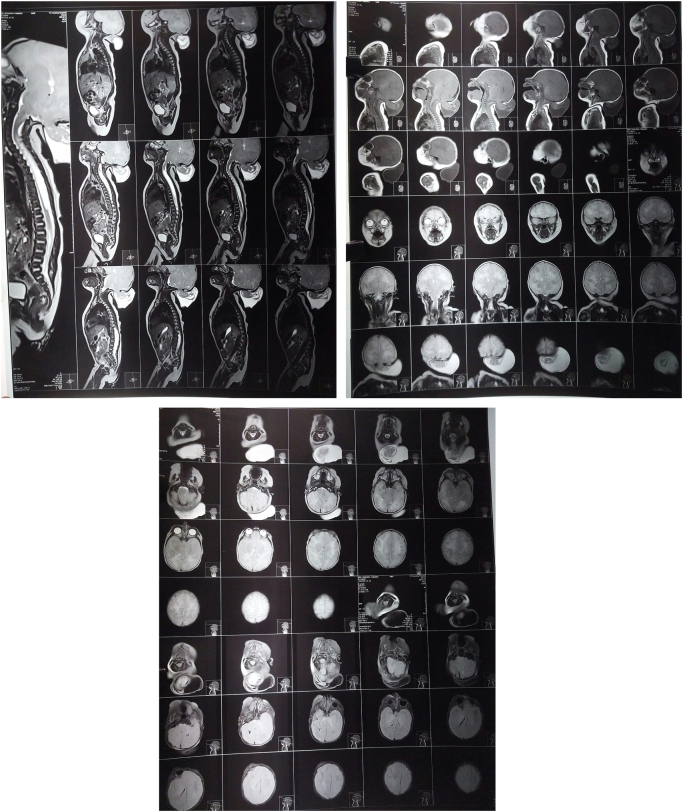
Imaging reveals an occipital calvarial defect measuring 18 × 18 mm, with herniation of the meninges, bilateral occipital lobes, and cerebellar vermis forming a cystic structure measuring 7.4 × 6.2 cm.

**Figure 3.**
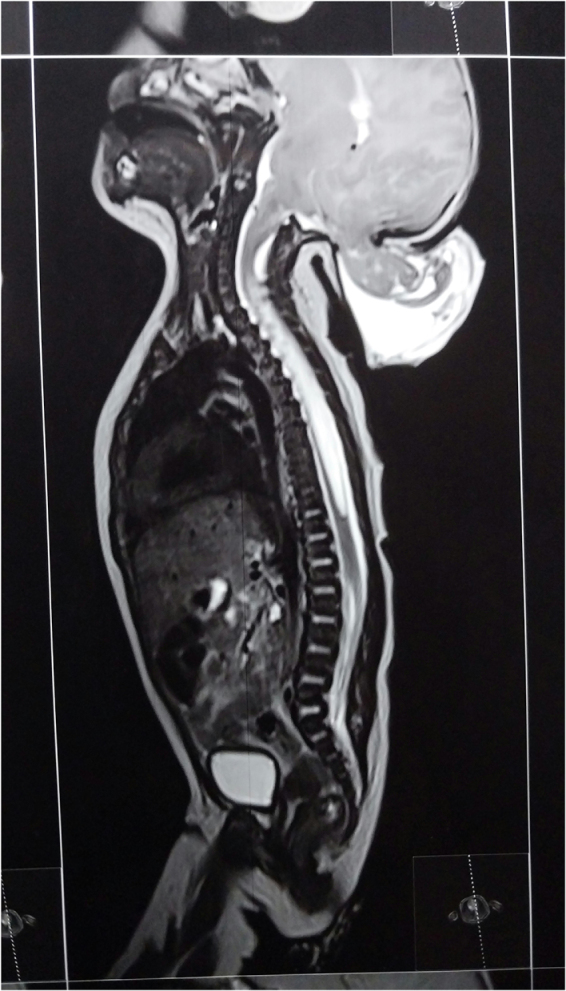
T2-weighted MRI showing a syrinx extending from C5–C6 to L1–L2 with mild cord expansion with maximum diameter is 8.1 mm and syrinx-to-cord ratio of 8.1:13.

There was an occipital calvarial defect measuring 18 × 18 mm. Through this, there was herniation of the meninges and bilateral occipital lobes and cerebellar vermis forming a cystic structure measuring 7.4 × 6.2 cm (Fig. 2). There was inferior descent of the obex and cerebellar tonsils below the basion-opisthion line, with resultant crowding of the foramen magnum. The fourth ventricle was partially effaced, but no hydrocephalus was observed. There was also effacement of the cortical sulci. There was a T2 high signal intensity syrinx extending from the C5–C6 to L1–L2 vertebral level, causing mild cord expansion and thinning of the cord. The maximum diameter of the syrinx measured 8.1 mm in caliber. The syrinx-to-cord ratio measured 8.1:13 (Fig. 3).

Surgery was planned to excise and repair the occipital encephalocele. Under strict aseptic conditions, the patient was placed in a prone position with her head secured in a Mayfield horseshoe headrest. An elliptical incision was given; the sac was opened, CSF was drained, dissection was continued until reaching the bony edge, herniated cerebellum was resected, and dura was closed in water tight fashion. The total duration of the surgery was approximately 2 hours. Following surgery, the infant was monitored in the Pediatric Intensive Care Unit for 1 day and then transferred to the general ward. She continued to feed well and moved all four limbs normally.

## Case discussion

CM III is the rarest and most severe form among the Chiari malformations, first described by Hans Chiari in 1891. It is characterized by herniation of posterior fossa contents – including the cerebellum, brainstem, and occasionally occipital lobes – through a high cervical or low occipital encephalocele. An encephalocele is a condition characterized by the protrusion of brain tissue through a defect in the skull. Unlike Chiari malformation types I and II, which are more commonly encountered, CM III accounts for <1% of all Chiari cases, with few cases reported in the literature to date^[7,8]^.

The etiology of CM III is not precisely known, but it is assumed that the formation of an imperfect occipital area due to incorrect neuralization during the ventricular extension process in the embryo causes prolapse of the cerebellum and the brainstem^[9]^. Some theories suggest that a lack of proper distension of the embryonic ventricular system causes posterior fossa hypoplasia, leading to brain tissue displacement and herniation^[10]^. In our case, the mother did not take folate during pregnancy, which might have led to a neural tube defect and subsequently encephalocele.

Clinically, CM III presents at birth or in the early neonatal period, often with a visible encephalocele or myeloencephalocele and varying degrees of neurological impairment. The brain structures most frequently herniating into the sac, in descending order of occurrence, are the cerebellum, followed by the occipital lobe, and then the parietal lobe^[7]^. Associated anomalies are common and may include hydrocephalus, microcephaly, agenesis of the corpus callosum, tethered cord syndrome, and spinal anomalies^[11–13]^. Neurological deficits depend on the extent and nature of the herniated neural tissue. Some patients may remain relatively stable, whereas clinical manifestations in others may include abnormal ocular movements such as titubation and downbeat nystagmus, along with neurological deficits like sensory impairment, muscle weakness, and ataxia. Respiratory complications range from insufficiency to failure. Additional features may encompass amyotonia, hyperreflexia, dysphagia leading to aspiration, variations in muscle tone – either spasticity or hypotonia – and inspiratory stridor^[7,11]^.

Our patient had swelling over the occiput, irregular in shape, smooth, with thin overlying skin. Among reported CM III cases, our case is unique due to the co-occurrence of dysmorphic features including microcephaly, low-set ears, a depressed nasal bridge, and a bluish spot on the dorsum of the right foot – suggestive of a syndromic association. While CM III typically presents with isolated CNS defects, its coexistence with craniofacial dysmorphisms raises suspicion for an underlying genetic or chromosomal syndrome, though definitive genetic testing was not available in this case.

Prenatal morphological ultrasound may reveal abnormalities like a cystic cervical mass, enlarged cerebral ventricles (ventriculomegaly), or underdeveloped brain structures such as microencephaly. Advances in imaging have shown that fetal MRI offers superior delineation of intracranial anomalies, especially those involving the posterior fossa, enhancing diagnostic precision before birth^[14]^. Postnatal MRI is pivotal for diagnosis. It not only delineates the contents of the encephalocele sac but also identifies associated intracranial and spinal anomalies. Typical findings include herniation of the cerebellum, followed by the occipital lobe, and then the parietal lobe. Occasionally, components within the encephalocele sac are necrotic, gliotic, or consist of heterotopic tissue or meningeal fibrosis, making them suitable for excision without causing additional neurological deficits^[15]^. There may also be absence or hypoplasia of posterior fossa structures, and possible ventriculomegaly^[7,16]^.

In a retrospective analysis by Castillo *et al* involving nine neonates with CM III, high cervical or low occipital encephaloceles were consistently noted, with variable herniation of brain tissues. Most commonly, the cerebellum and occipital lobes were present within the sac, followed by the fourth and lateral ventricles, and in rare instances, brainstem components such as the medulla and pons^[17]^.

Before surgery, to prevent massive bleeding, angio-MRI is important for localizing venous sinuses and assessing their patency^[5]^. In our patient, we performed MRI which showed inferior descent of the cerebellar tonsils and obex with occipital encephalocele. This suggests CM III. There was also a syrinx in the spinal cord from C5 to L2. Additionally, echocardiography suggested a patent foramen ovale with left-to-right shunting. The prognosis of CM III was historically considered poor, with high rates of mortality and severe disability. However, advancements in neurosurgical techniques have improved outcomes in select cases. Surgical intervention is the primary treatment for meningoencephalocele in patients with CM III, aiming to prevent complications such as encephalocele rupture or the development of meningitis^[5]^.

The decision to perform surgery, as well as its timing, depends on several factors, such as the condition of the skin covering the encephalocele, the volume of herniated neural tissue, the presence of vital structures within the sac, and the extent of neurological deficits^[18]^. Encephaloceles are typically treated on an elective basis; however, if a rupture occurs, prompt surgical intervention is necessary due to the risk of central nervous system infection from exposure of the meninges and brain tissue^[19]^. Surgical intervention typically involves repair of the encephalocele, dural closure, and management of hydrocephalus (if present) with ventriculoperitoneal shunting^[11,16,20]^. Favorable outcomes have been reported in patients where herniated neural tissue was non-functional or where surgery was performed early and meticulously^[20]^. The encephalocele in our patient ruptured, for which excision and repair of the encephalocele were performed. Ventriculoperitoneal shunting was not needed.

## Conclusion

CM III is a rare and severe congenital hindbrain anomaly characterized by herniation of brain tissue through an occipital or cervical encephalocele, often leading to significant neurological impairment and a poor prognosis. Early diagnosis through imaging and timely surgical intervention, especially in cases of encephalocele rupture, are critical to prevent complications such as infection and neurological deterioration. Advances in neurosurgery have improved outcomes, but management remains challenging due to the complexity and severity of the malformation.

## Data Availability

The data that support the findings of this study are available from the corresponding author upon reasonable request.

## Research registration unique identifying number (UIN)

Case reports do not need to be registered.

## References

[1] Tubbs S Turgut M Oakes WJ The Chiari Malformations.

[2] Fisahn C Shoja MM Turgut M. The chiari 3.5 malformation: a review of the only reported case. Child’s Nerv Syst 2016;32:2317–19. 27679454 10.1007/s00381-016-3255-3

[3] Pang D Dias MS. Cervical myelomeningoceles. Neurosurgery 1993;33:363–73. 8413865 10.1227/00006123-199309000-00003

[4] Cama P Piatelli GL Fondelli MP. Chiari complex in children - neuroradiological diagnosis, neurosurgical treatment and proposal of a new classification (312 Cases). Eur J Pediatr Surg 1995;5:35–38. 8770577 10.1055/s-2008-1066261

[5] Jeong DH Kim CH Kim MO. Arnold-Chiari malformation type III with meningoencephalocele: a case report. Ann Rehabil Med 2014;38:401–04. 25024966 10.5535/arm.2014.38.3.401 PMC4092183

[6] Ahmed K Ahmed A Ginimol M. Revised Surgical CAse REport (SCARE) Guideline: an update for the age of Artificial Intelligence. Prem J Sci 2025;2.

[7] Ivashchuk G Loukas M Blount JP. Chiari III malformation: a comprehensive review of this enigmatic anomaly. Child’s Nerv Syst 2015;31:2035–40. 26255148 10.1007/s00381-015-2853-9

[8] Safari H. Chiari malformation Type III with good outcome: case-report and review of clinical and radiological findings. J Head Neck Spine Surg 2018;2:1–5.

[9] McLone DG Knepper PA. The cause of chiari II malformation: a unified theory. Pediatr Neurosurg 2008;15:1–12. 10.1159/000120432 2699756

[10] Swaiman KF Ashwal S Ferriero DM. Swaiman’s Pediatric Neurology E-Book: Principles and Practice. 6th ed. Elsevier Health Sciences, Philadelphia; 2017.

[11] Caldarelli M Rea G Cincu R. Chiari type III malformation. Child’s Nerv Syst 2002;18:207–10. 12042918 10.1007/s00381-002-0579-y

[12] Ortiz JF Ruxmohan S Alli A. Chiari malformation type III: a case report and review of literature. Cureus 2021;13:e14131. 33912363 10.7759/cureus.14131 PMC8071580

[13] Işik N Elmaci I Silav G. Chiari malformation type III and results of surgery: a clinical study: report of eight surgically treated cases and review of the literature. Pediatr Neurosurg 2009;45:19–28. 19221459 10.1159/000202620

[14] Mekouar Y Laoudiyi D Haboussi MR. Chiari type III malformation: case report and review of literature. Radiol Case Rep 2022;17:628–30. 35027985 10.1016/j.radcr.2021.11.063 PMC8715142

[15] Ramdurg SR Solpure S Dubey S. Asymptomatic Chiari III malformation with tectal beaking and holocord syrinx. J Pediatr Neurosci 2013;8:254. 24470829 10.4103/1817-1745.123702 PMC3888052

[16] Halefoglu AM Halefoglu AM Yilmaz A. Case Report chiari type III malformation presenting with two huge encephaloceles in a new born infant: case Report. Acta Med Iran 2023;61:57–60.

[17] Castillo M Quencer RM, and Dominguez R. Chiari III malformation: imaging features [Internet]. AJNR Am J Neuroradiol 2025;13:107–13. PMC8331797 1595427

[18] Bulut MD Yavuz A Bora A. Chiari III malformation with a giant encephalocele sac: case report and a review of the literature. Pediatr Neurosurg 2013;49:316–19. 25277137 10.1159/000365661

[19] Sharma M Sherwani P Venkataram T. Intraventricular pneumocephalus: ruptured occipital encephalocele. Indian J Pediatr 2022;89:722. 35353364 10.1007/s12098-022-04149-5

[20] Elbaroody M Mostafa HE Alsawy MFM. Outcomes of chiari malformation III: a review of literature. J Pediatr Neurosci 2020;15:358. 33936298 10.4103/jpn.JPN_135_19 PMC8078634

